# Supplementary material for: Interferon-α promotes HLA-B-restricted presentation of conventional and alternative antigens in human pancreatic β-cells
Source: Nat Commun. 2025 Jan 17;16:765. doi: 10.1038/s41467-025-55908-9 (PMC11748642; doi:10.1038/s41467-025-55908-9)
Supplement: Supplementary file 1 — Supplementary Information [file 41467_2025_55908_MOESM1_ESM.pdf]

# Interferon-α promotes HLA-B-restricted presentation of conventional and alternative antigens in human pancreatic β-cells

Alexia Carré et al.

## Supplementary Information

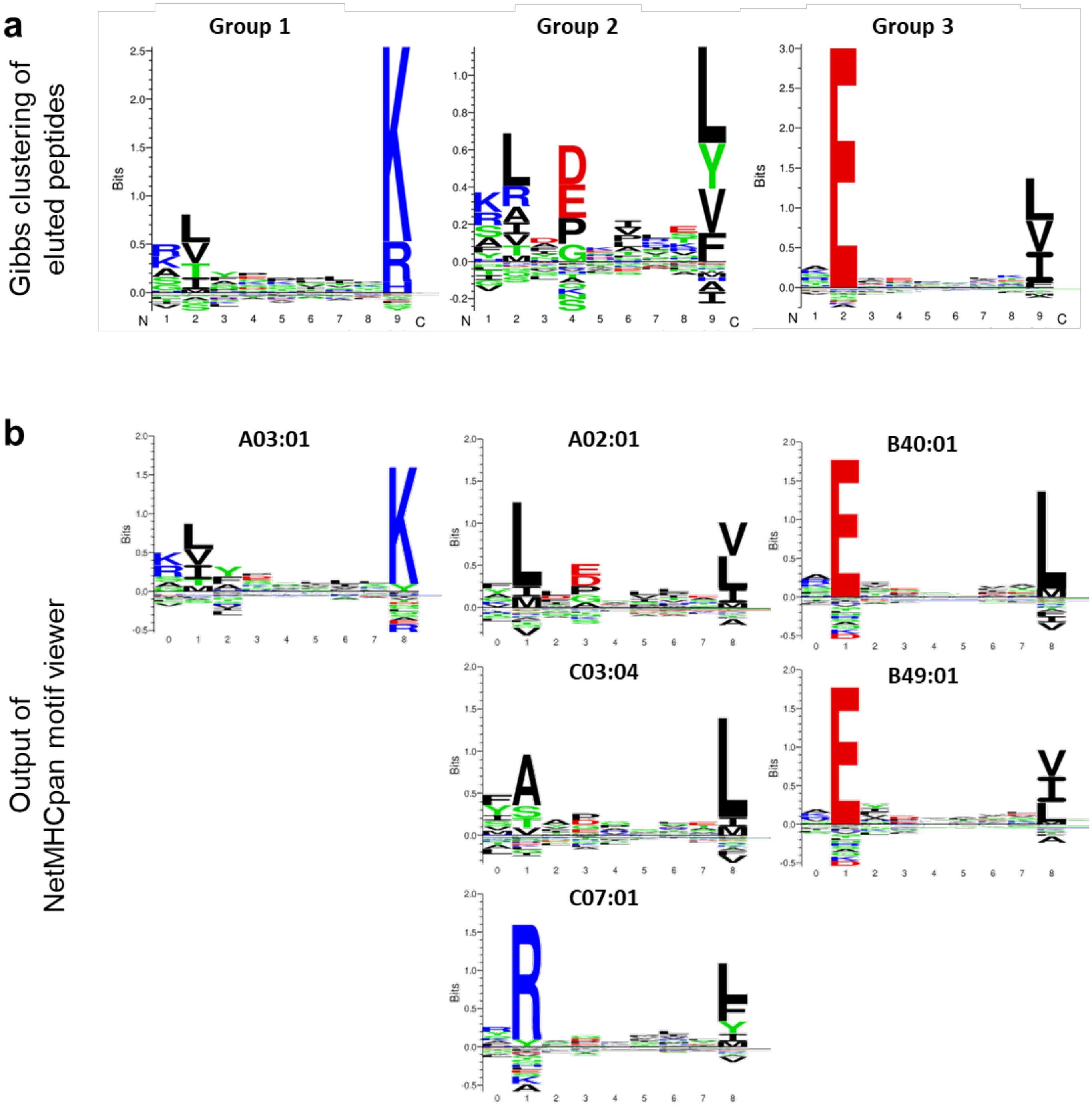


**Supplementary Fig. 1. Gibbs clustering of 8-14mer peptides. a** All unique conventional and post-translationally modified candidates identified in the 8-14 aa length range were clustered using GibbsCluster. The *x*-axis indicates the residue position within the 9mer core sequence. Each aa is represented by its single-letter code, with its size proportional to its frequency at the indicated position. All peptides were inputed as unmodified sequences. **b** NetMHCpan4.1a sequence motifs for the HLA-I alleles expressed by the ECN90 β-cells.

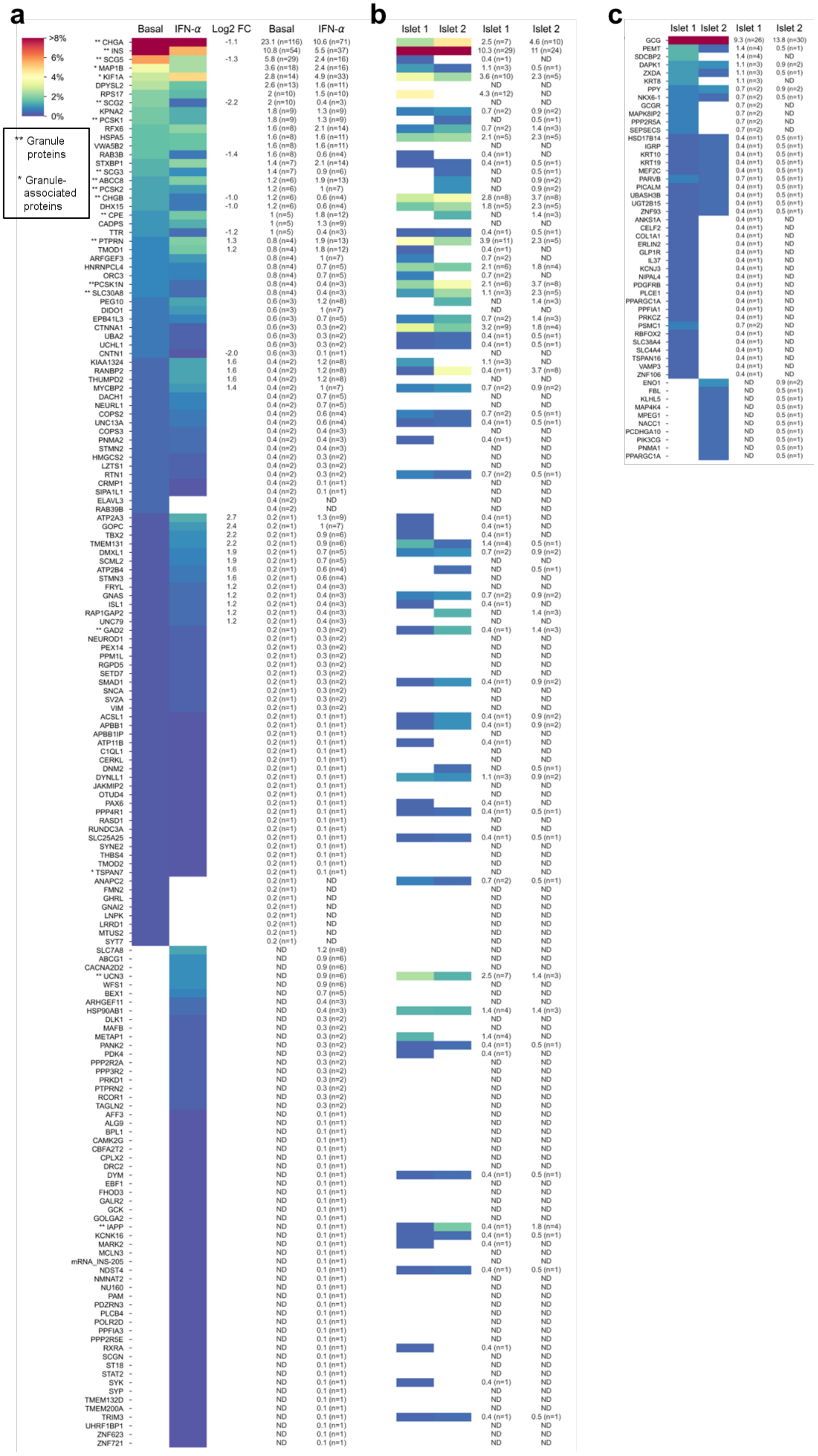
a
Basal
IFN-α
Log2 FC
Basal
IFN-α
>8%
6%
4%
2%
0%
** Granule proteins
* Granule-associated proteins
b
Islet 1
Islet 2
Islet 1
Islet 2
c
Islet 1
Islet 2
Islet 1
Islet 2

**Supplementary Fig. 2. Heatmap of the source proteins of the immunopeptidome of ECN90 β-cells and primary human islets. a-b** Relative representation of source proteins in ECN90 β-cells (a) and human islets (b), along with the corresponding significant log2 fold changes (FC) and percent values, ranked according to the number of peptides detected in the IFN-α-treated condition. **c** Relative representation of GCG and β-cell-enriched source proteins in two different primary islet preparations. In all panels, relative representation of each source protein is calculated based on the number of unique peptides identified for each protein out of the total number of peptides in a given condition, expressed as percentage. Ligands included herein are conventional, post-translationally modified and mRNA splice peptides. Peptides carrying PTMs were counted only for PTMs defined as likely biological; they were otherwise counted as unmodified. mRNA splice variants were considered only when validated by spectral matching.

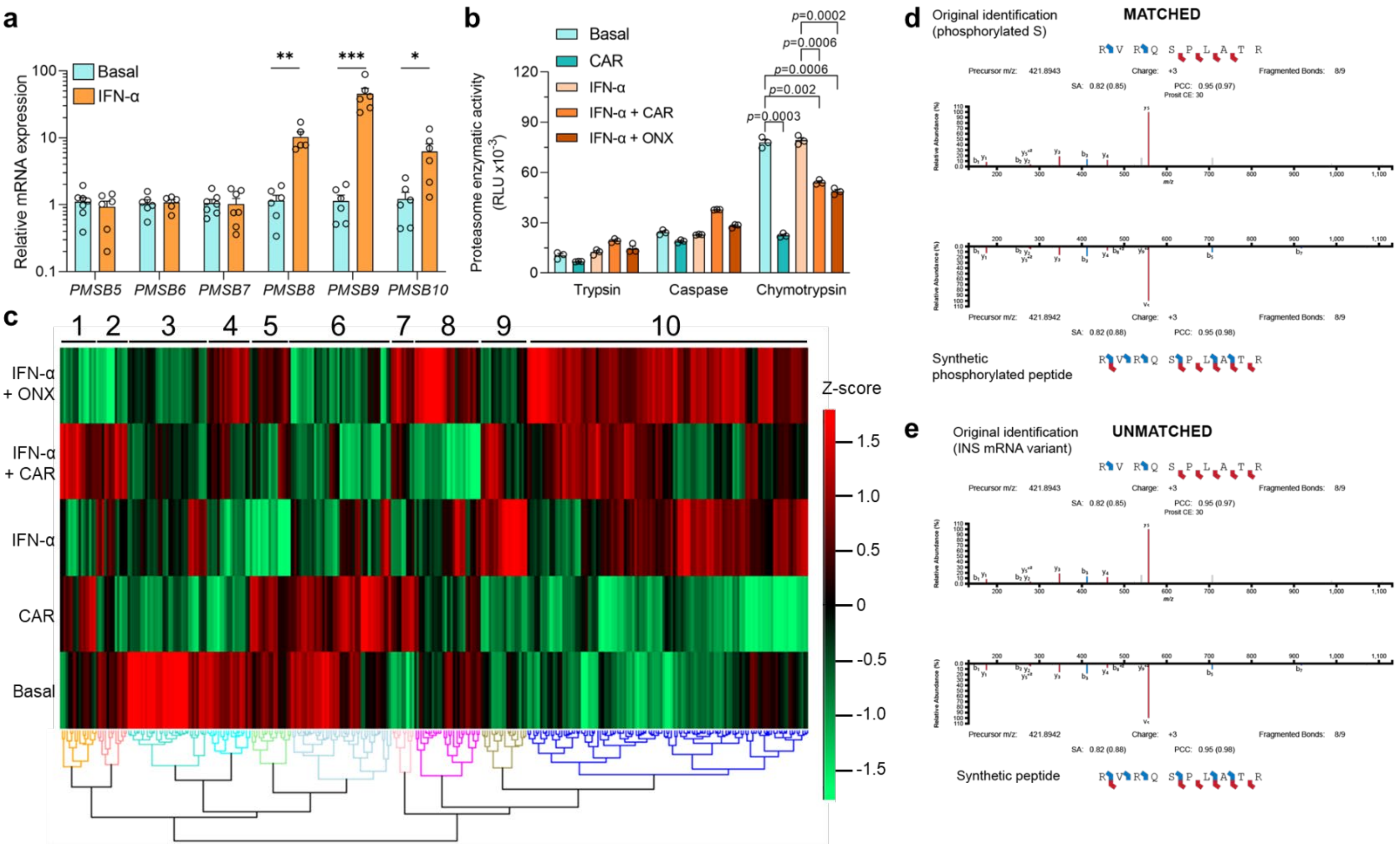


**Supplementary Fig. 3. Inhibition of constitutive and immuno-proteasome in ECN90 β-cells. a** Gene expression of the subunits of the constitutive (left; *PSMB5*, *PSMB6*, *PSMB7*) and immuno-proteasome (right; *PSMB8*, *PSMB9*, *PSMB10*) in ECN90 β-cells assessed by qPCR (mean+SEM of 6 biological replicates). *$p$=0.015, **$p$=0.004, ***$p$=0.002 by Mann-Whitney U test. **b** Enzymatic activity of the three proteasome catalytic subunits in basal and IFN-α-treated ECN90 β-cells (3-5×$10^6$) with or without carfilzomib (CAR; constitutive proteasome inhibitor) or ONX-0914 (ONX; immunoproteasome inhibitor). Data represent mean+SEM of triplicate measurements from a representative experiment out of 3 performed; $p$ values calculated by Student's t test. **c** Heatmap of HLA-I-eluted peptides identified in the indicated conditions. For each precursor found in ≥2 replicates in one of the 5 conditions, ion intensities were summed. For peptides identified in multiple instances, median values were computed. The values were $\log_2$-transformed, missing values were imputed from normal distribution downshifted by 1.8 and normalization was computed using row z-scores. The heatmap was generated with Perseus (v2.0.6), clustering rows with "average" as the agglomeration method and "Pearson correlation" as the distance matrix. Dendrogram colors on the bottom and numbers on top indicate distinct peptide clusters (see Results). The identity of peptides in each cluster is provided in Supplementary Data 7. **d-e** Spectral matching examples of matched (d) and unmatched (e) synthetic peptides compared to the initial peptide identification using the online tool available at https://www.proteomicsdb.org/use.

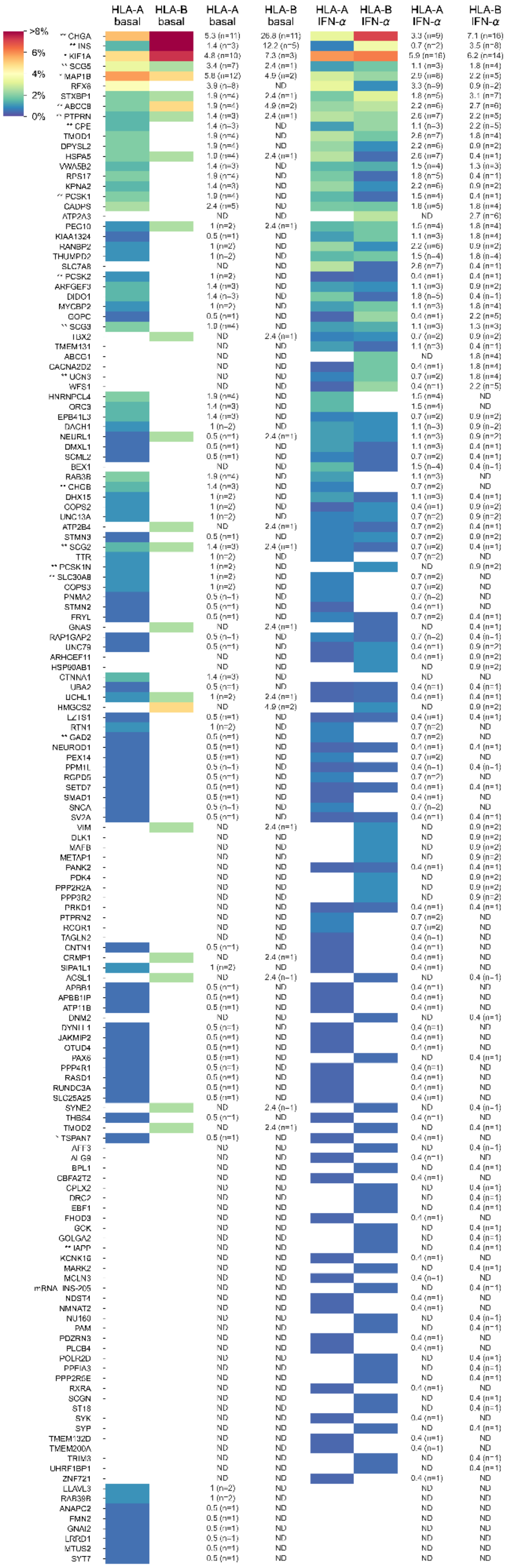


**Supplementary Fig. 4.** Heatmap of the source proteins of the immunopeptidome of ECN90 β-cells separated by predicted HLA-A and HLA-B restriction. Ranking and legend is the same as in Supplementary Fig. 2.

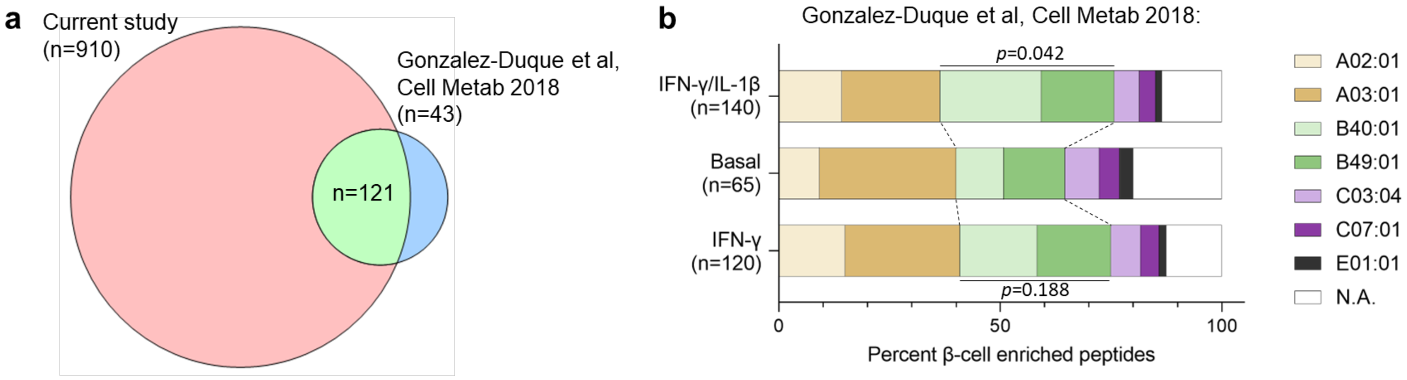


**Supplementary Fig. 5. Comparison between the current immunopeptidomics dataset and the dataset generated in Gonzalez-Duque et al, *Cell Metab* 2018. a** Peptide yield comparison between the two studies. **b** Relative distribution of predicted HLA-I ligands for each allele expressed by ECN90 β-cells in basal and cytokine-treated conditions from the previous dataset using the current bio-informatics pipeline. The indicated $p$ values were calculated by Fisher exact test.

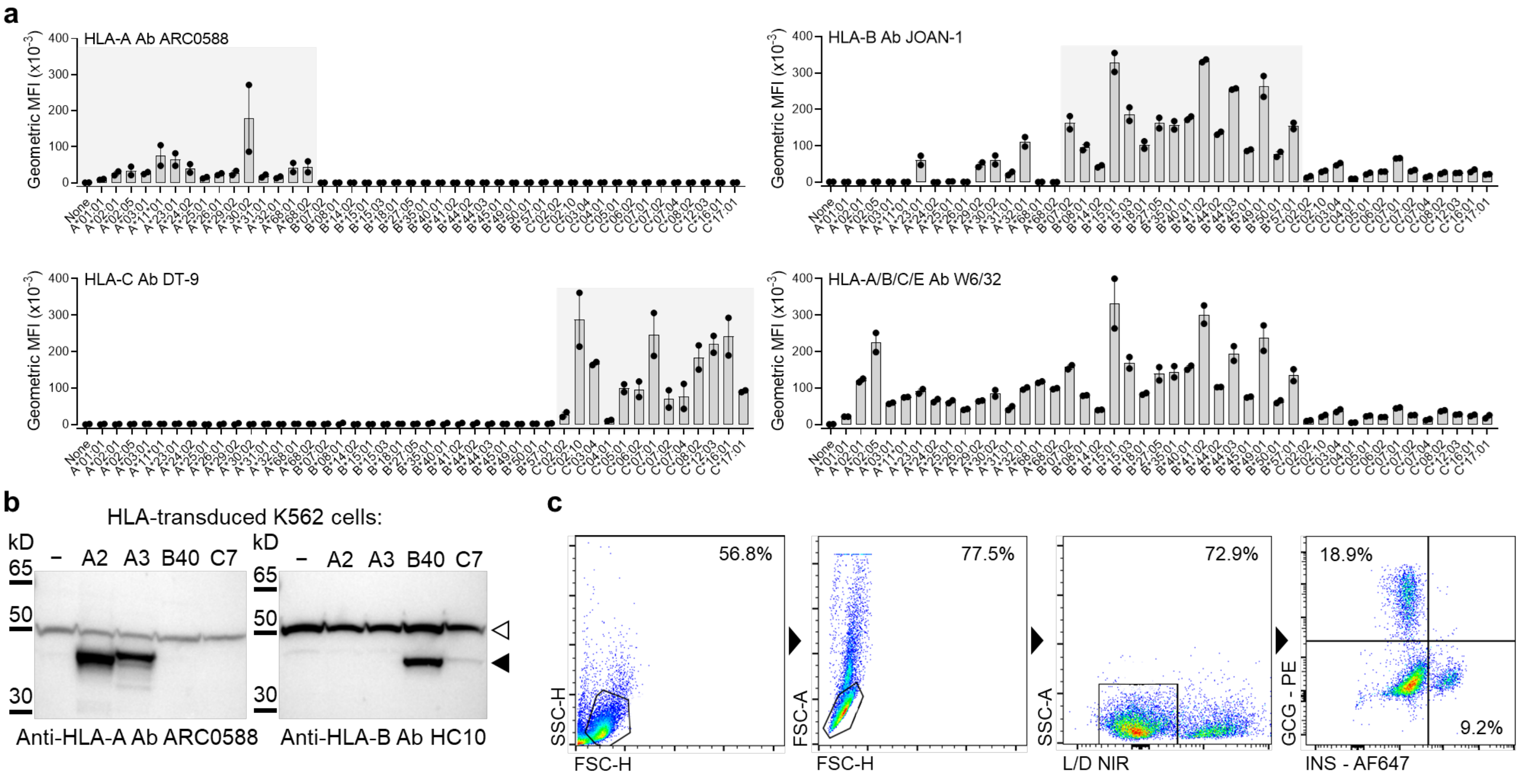


**Supplementary Fig. 6. Validation of the specificity of Abs recognizing HLA-A, -B or -C. a** Flow cytometry staining of HLA-I$^{-}$ K562 cells transduced with the indicated HLA-I alleles. Data represent mean±SEM of duplicate measurements from a representative experiment out of 2 performed. **b** Western blot detection of HLA-I heavy chains (black arrowhead) with the indicated Abs on HLA-I$^{-}$ K562 cells transduced with the indicated HLA-I alleles. α-tubulin was used as loading control (white arrowhead). A representative experiment out of 3 performed is shown. **c** Gating strategy for the analysis of HLA-A, -B and -C expression in primary human islets (presented in Fig. 6b).

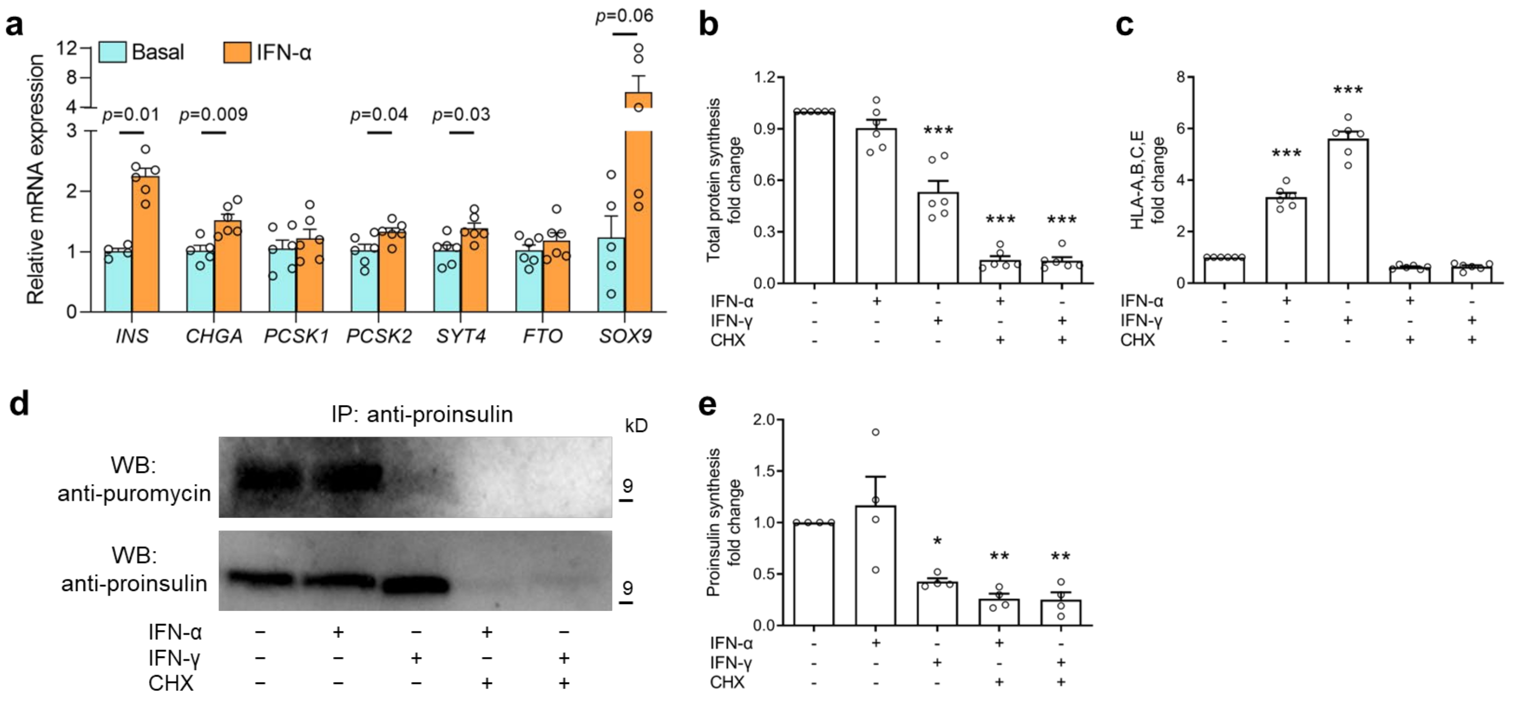


**Supplementary Fig. 7. IFN-α does not induce dedifferentiation or decreased proINS synthesis. a** Relative mRNA expression of β-cell identity genes in ECN90 β-cells exposed or not to IFN-α. *PPIA* was used as internal normalizing control, and each gene was normalized to the basal sample. Data represent mean+SEM of 6 biological replicates. The indicated *p* values were calculated by Mann-Whitney U test. **b-c** Puromycin incorporation in newly synthesized total proteins (b) and HLA-A/B/C/E expression (c), detected by flow cytometry and expressed as fold change compared to the basal sample (first column). Inhibition of protein synthesis by cycloheximide (CHX) provided negative controls (last 2 columns). Data represent mean+SEM of 6 biological replicates. ***$p$<0.0001 by one-way ANOVA. **d** Puromycin incorporation in newly synthesized proINS, detected by proINS immunoprecipitation (IP) followed by Western blot (WB) for puromycin (top; corresponding to newly synthesized proINS) and proINS (bottom; corresponding to total proINS). A representative experiment out of 4 performed is shown. **e** Puromycin incorporation in newly synthesized proINS, detected by Western blot and expressed as fold change compared to the basal sample (first column). Data represent mean+SEM of 4 biological replicates. *$p$=0.03 and **$p$=0.002 by one-way ANOVA.

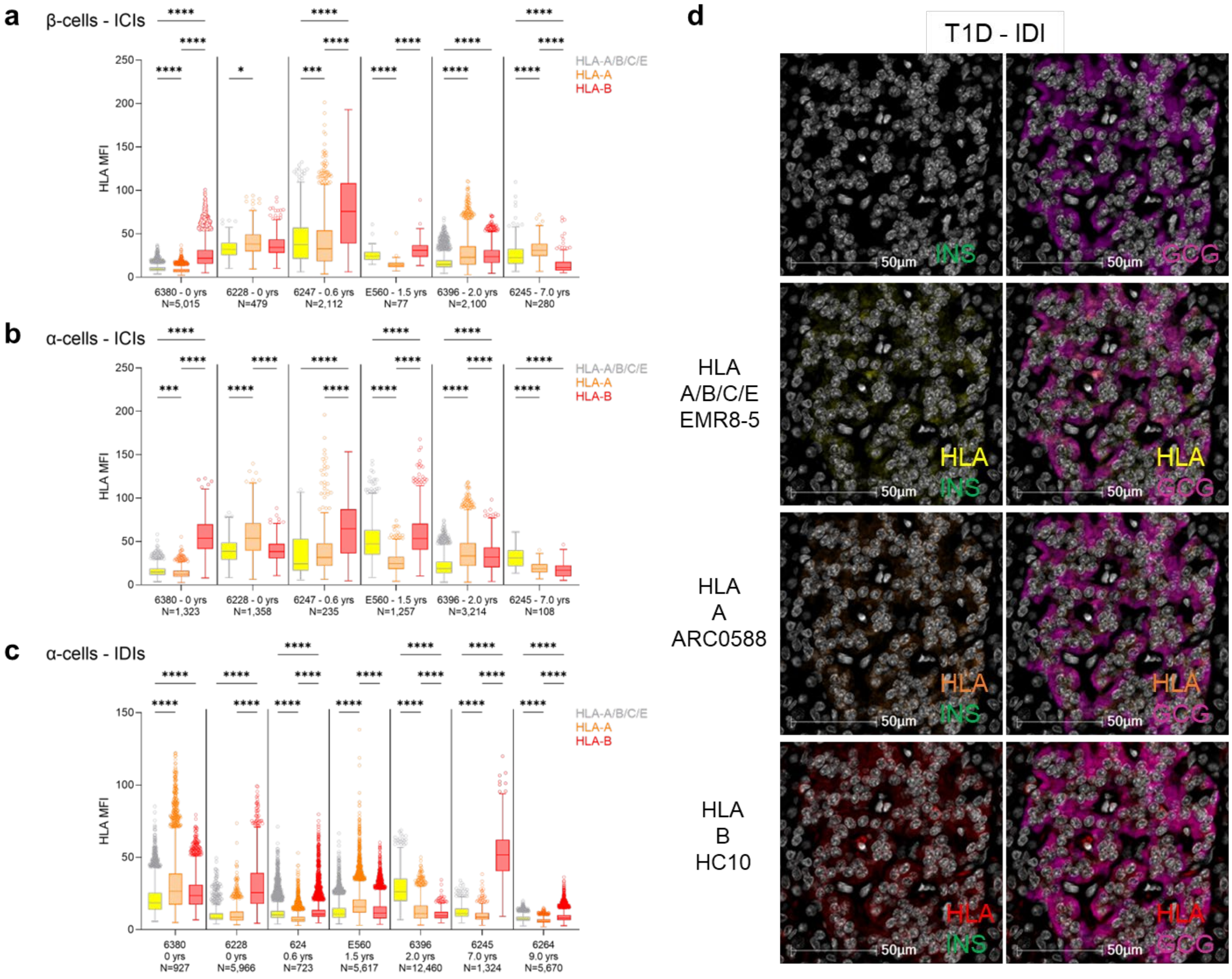

**Supplementary Fig. 8. HLA-B vs. HLA-A hyper-expression in the islets of individual T1D cases. a-b-c** Immunofluorescence quantification of HLA-I mean fluorescence intensity (MFI) for HLA-A/B/C/E (yellow/grey), HLA-A (orange) and HLA-B (red) in individual β-cells (a), insulin-containing islet (ICI) α-cells (b) and insulin-deficient islet (IDI) α-cells (c) from each T1D case (listed in Supplementary Table 2). T1D duration and number of cells analyzed are indicated for each donor. Boxes depict median and interquartile range values, with whiskers and outliers plotted with the Tukey method. Case nPOD 6264 did not display any ICI and was therefore analyzed only for IDIs. **p*=0.04, ****p*=0.0006 and *****p*<0.0001 (panel a), ****p*=0.0008 and *****p*<0.0001 (panel b), *****p*<0.0001 (panel c) by Dunn's multiple comparison test. **d** Representative immunofluorescence images of IDIs from T1D case nPOD 6396, stained for INS (green, first column) or GCG (violet, second column), alone (first raw) or in combination with HLA-A/B/C/E (yellow; second raw), HLA-A (orange; third raw) and HLA-B (red, fourth raw). Scale bar 50 µm.

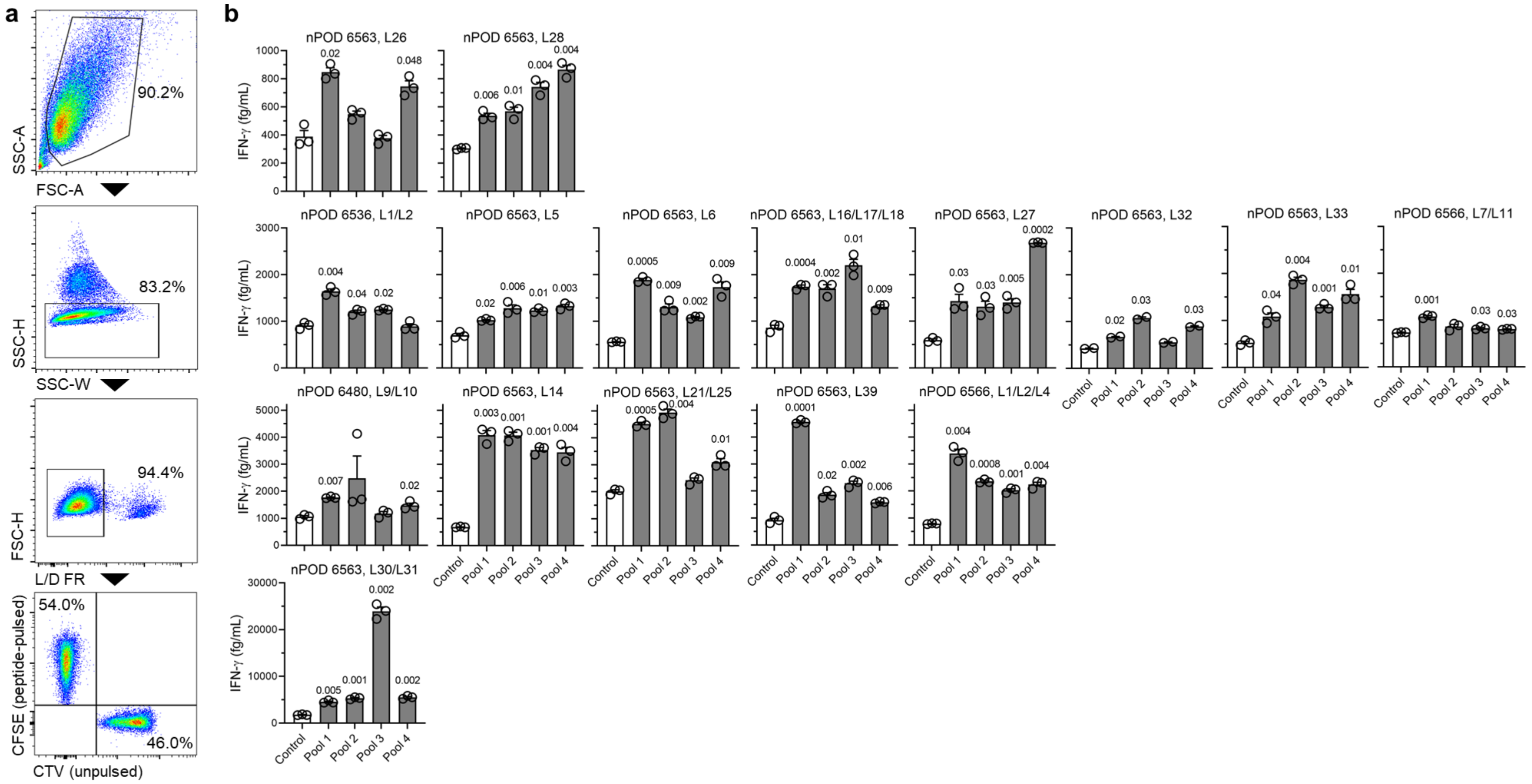


**Supplementary Fig. 9. Gating strategy for β-cell cytotoxicity assays and recognition of HLA-B40-restricted peptide pools in the islets of T1D donors. a** Gating strategy used for ECN90 β-cell cytotoxicity assays (presented in Fig. 8d); see Methods for details. **b** IFN-γ secretion by polyclonal $CD8^+$ T-cell lines expanded from islet infiltrates of $HLA\text{-}B40^+$ nPOD T1D donors (listed in Supplementary Table 3) and exposed to HLA-B40-transduced K562 antigen-presenting cells pulsed with HLA-B40-restricted peptide pools (listed in Supplementary Table 4). Data represent mean+SEM of triplicate measurements from a representative experiment out of 2 performed; *p* values by paired Student's t test indicated on top of bars. Three additional T-cell lines responding to HLA-B40-restricted peptide pools and deconvoluted for individual peptide reactivities are presented in Fig. 9.

```
  MKSIYFVAGL FVMLVQGSWQ RSLQDTEEKS RSFSASQADP LSDPDQMNED KRHSQGTFTS DYSKYLDSRR AQDFVQWLMN
  MKSIYFVAGL            RSLQDTEEKS R                    HSQGTFTS D          AQDFVQWLMN ►
   KSIYFVAGL            RSLQDTEEKS RSF                  HSQGTFTS DY           DFVQWLMN ►
                         SLQDTEEKS                      HSQGTFTS DYSK          FVQWLMN ►
                         SLQDTEEKS RS                      GTFTS DYSK
                         SLQDTEEKS RSFS                      FTS DYSKYL
                          LQDTEEKS RSFS                       TS DYSKYL
                            DTEEKS RSFS                       TS DYSKYLDS
                            DTEEKS RSFSA                       S DYSKYLD
                            DTEEKS RSFSAS                      S DYSKYLDS
                             TEEKS RSFSA                         DYSKYLDS
                                    SFSASQAD                      YSKYLDSRR AQD
                                     FSASQADP L
                                            P LSDPDQMNE

  TKRNRNNIAK RHDEFERHAE GTFTSDVSSY LEGQAAKEFI AWLVKGRGRR DFPEEVAIVE ELGRRHADGS FSDEMNTILD
◄ TKRN         HDEFERHAE G               AAKEFI AWL
◄ T            HDEFERHAE GT                 EFI AWLVKG
◄ T               FERHAE GTFT                FI AWLVKG
                  FERHAE GTFTSD
                  FERHAE GTFTSDVS
                      AE GTFTSDV
                           FTSDVSSY L

NLAARDFINW LIQTKITDRK
      FINW LIQTKITD
       INW LIQTKITD
           LIQTKITD
```

Signal peptide
GRPP
IP-1
Major proGCG fragment

**Supplementary Fig. 10. Mapping of the GCG peptides eluted from HLA-I molecules of primary islet samples.** These peptides are detailed in Supplementary Data 5. GRPP, glicentin-related pancreatic polypeptide; IP-1, intervening peptide-1.

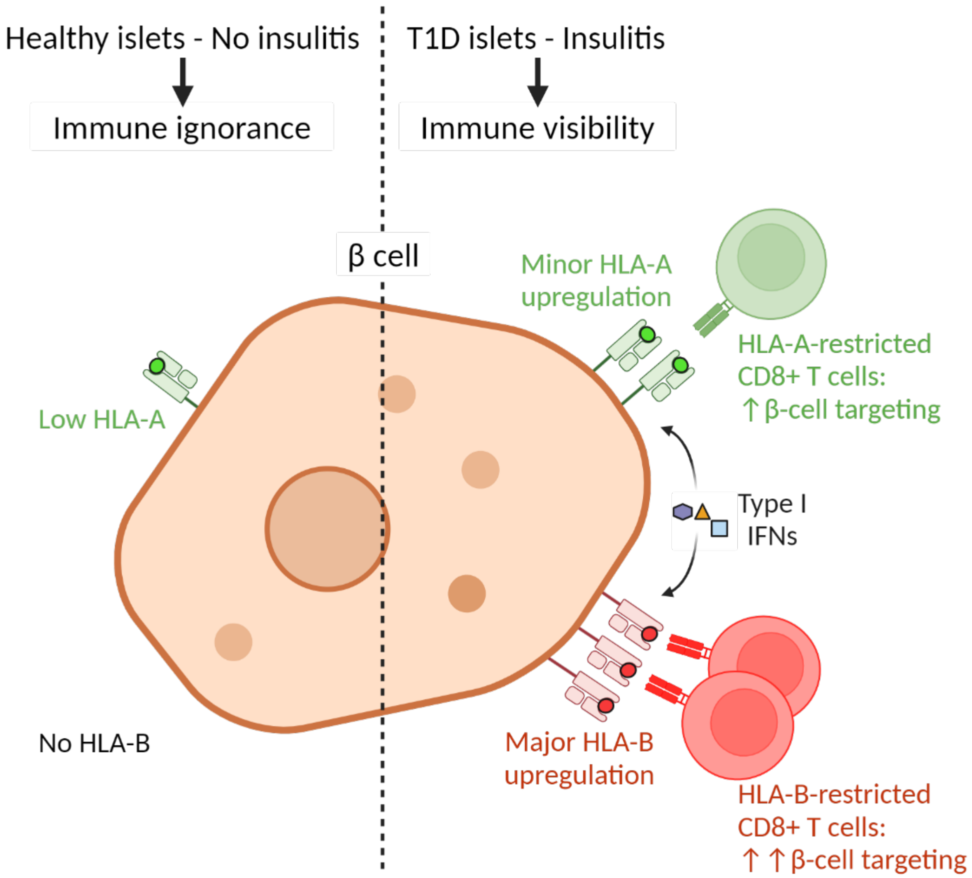


**Supplementary Fig. 11. Model of preferential HLA-B upregulation in the insulitis microenvironment.** In the healthy state (left), immune ignorance is maintained by the low expression of HLA Class I, mainly HLA-A, by β-cells. In the T1D state (right), the type I IFN signature of insulitis leads to the upregulation of HLA Class I, mainly HLA-B, which favors the cytotoxic targeting of β-cells by HLA-B-restricted $CD8^+$ T cells. Created in BioRender. Mallone, R. (2025) https://BioRender.com/v47f856.

| Sample | RRID | Purity | Viability | Sex | Age (yrs) | BMI ($kg/m^2$) | HbA1c | HLA-A | HLA-B |
|---|---|---|---|---|---|---|---|---|---|
| 1 | SAMN39911708 | 75% | 97% | F | 42 | 26 | 5.7% | A1, A2 | B7, B8 |
| 2 | SAMN40619409 | 90% | 95% | M | 63 | 34 | 5.1% | A2, A68 | B7, B35 |
| 3 | SAMN41142257 | 90% | 95% | F | 39 | 37 | 6.0% | A2, A68 | B35, B52 |
| 4 | SAMN41299360 | 85% | 93% | M | 20 | 24 | 5.1% | A2, A2 | B60, B44 |
| 5* | SAMN43474066 | 95% | 95% | M | 54 | 22 | 5.3% | A2, A2 | B35, B35 |
| 6* | SAMN43551039 | 90% | 95% | M | 42 | 34 | 5.4% | A2, A29 | B44, B45 |
| 7* | SAMN43762145 | 90% | 95% | M | 53 | 33 | 5.6% | A2, A23 | B8, B44 |

**Supplementary Table 1. Primary human islet samples analyzed for the expression of HLA-A, -B and -C.** All samples were from non-diabetic donors and were analyzed by flow cytometry (Fig. 6b); samples indicated with an asterisk were also analyzed by RT-qPCR (Fig. 6a).

| Donor group | nPOD/EADB case RRID | Sex | Age (yrs) | T1D (yrs) | Positive Auto-Abs | C-peptide (ng/mL) | HLA-A | HLA-B |
|---|---|---|---|---|---|---|---|---|
| T1D | nPOD 6228 SAMN15879284 | M | 13 | 0 | GAD/IA-2 ZnT8 | 0.10 | A*23:01 A*68:01 | B*44 B*60 |
| T1D | nPOD 6245 SAMN15879301 | M | 22 | 7.0 | GAD/IA-2 | <0.05 | A*03:01 A*32:01 | B*61 B*65 |
| T1D | nPOD 6247 SAMN15879303 | M | 24 | 0.6 | mIAA | 0.47 | A*24:02 A*68:01 | B*44 B*60 |
| T1D | nPOD 6264 SAMN15879318 | F | 12 | 9.0 | None | <0.05 | A*23:01 A*32:01 | NA |
| T1D | nPOD 6380* SAMN15879433 | F | 12 | 0 | None | 0.22 | A*33:03* A*68:02* | B*53 B*71 |
| T1D | nPOD 6396 SAMN15879449 | F | 17 | 2.0 | None | 0.06 | A*23:01 A*24:02 | B*44 B*49 |
| T1D | EADB E560 | F | 42 | 1.5 | NA | NA | NA | NA |
| ND | nPOD 6160 SAMN15879216 | M | 22 | - | None | 0.40 | A*11 A*29 | B*35 B*44 |
| ND | nPOD 6227 SAMN15879283 | F | 17 | - | None | 2.75 | A*02:01 A*03:01 | B60 B62 |
| ND | nPOD 6232 SAMN15879288 | F | 14 | - | None | 19.50 | A*02:01 A*24:02 | B*07 B*44 |
| ND | nPOD 6278* SAMN15879332 | F | 12 | - | None | 4.54 | A*23:01 A*68:02* | B*45 B*71 |
| ND | nPOD 6416 SAMN15879469 | M | 12 | - | None | 15.33 | A*01:01 A*11:01 | B*07 B*37 |
| ND | nPOD 6462* SAMN15879515 | F | 14 | - | None | 11.09 | A*02:01 A*25:01* | B*18:01 B*40:01 |

**Supplementary Table 2. nPOD and EADB cases analyzed for the expression of HLA-I.** Pancreas tissue immunofluorescence images and their analyses are presented in Fig. 7 and Supplementary Fig. 8. Asterisks indicate cases harboring HLA-A allotypes potentially cross-reactive with the HLA-B Ab HC10, indicated with crossed symbols in Fig. 7d. ND, not determined; NA, not available.

| nPOD case RRID:SAMN# | Sex | Age (yrs) | T1D (yrs) | Positive Auto-Abs | HLA-A | HLA-B | HLA-C | Positive/tested TCRs | Positive/tested T-cell lines |
|---|---|---|---|---|---|---|---|---|---|
| 6342<br>15879396 | F | 14 | 2 | IA-2 | 02:01<br>68:01 | **40:01**<br>**40:01** | 03:04<br>03:04 | 0/29 | NA |
| 6480<br>15879533 | M | 17 | 2.5 | IA-2 | 03:01<br>11:01 | 07:02<br>**40:01** | NA<br>NA | NA | 1/4 (pooled) |
| **6536**<br>**18242780** | F | 20 | 4.0 | GAD | 02:01<br>31:01 | 08:01<br>**40:01** | 03:04<br>07:01 | 1/28 | 1/3 (pooled)<br>0/3 (individual) |
| 6563<br>30386851 | F | 15 | 0 | IA-2 | 02:01<br>11:01 | **40:01**<br>50:01 | 03:04<br>06:02 | 0/20 | 5/5 (pooled)<br>9/10 (individual) |
| 6566<br>33284286 | M | 16 | 2.0 | GAD/IA-2<br>ZnT8 | 03:01<br>23:01 | **40:01**<br>50:01 | 03:04<br>06:02 | 0/24 | 3/5 (pooled)<br>0/1 (individual) |

**Supplementary Table 3. nPOD islet donors analyzed for the identification of HLA-B40-restricted TCRs and CD8$^+$ T cells.** For the identification of HLA-B40-restricted TCRs, TCRs from islet-infiltrating T cells of HLA-B40$^+$ nPOD donors were sequenced and re-expressed into ZsGreen fluorescent reporter 5KC T cells and tested with peptides listed in Supplementary Table 4. The nPOD case from whom TCR 173.D12 was selected is indicated in bold. For the identification of HLA-B40-restricted CD8$^+$ T cells, islet-derived T-cell lines from HLA-B40$^+$ nPOD donors were co-cultured with HLA-B40-transduced K562 antigen-presenting cells pulsed with the peptide pools listed in Supplementary Table 4. Pools of peptides binding to irrelevant HLA-I were used as negative controls (listed in Supplementary Table 5). Peptide recognition was assessed by measurement of IFN-γ secretion, as shown in Fig. 9. All T-cell lines tested responded to plate-bound anti-CD3/CD28 stimulation as positive control. The "Positive/tested T-cell lines" column displays the number of positive T-cell lines (tested in pools to reach required T-cell numbers, or individually when possible) out of those tested. NA, not available.

| Pool number | Peptide sequence | Source protein | AA position | Accession number | PTM | Positive samples | HLA restriction | NetMHCPan rank |
|---|---|---|---|---|---|---|---|---|
| **1** | **YEARFQQKL** | **ABCC8** | **1218-1226** | **Q09428** | **0** | **IFN-α** | **B40:01** | **0.012** |
| **1** | **LEFDKPEKL** | **ABCC8** | **1557-1565** | **Q09428** | **0** | **IFN-α** | **B40:01/B49:01** | **0.018/0.037** |
| **1** | **VEVISDTL** | **CHGA** | **37-44** | **P10645** | **0** | **IFN-α** | **B40:01/B49:01** | **0.357/0.838** |
| **1** | **KELQDLAL** | **CHGA** | **77-84** | **P10645** | **0** | **IFN-α** | **B40:01/B49:01** | **0.418/1.154** |
| **1** | **HSGFEDELSEVL** | **CHGA** | **97-108** | **P10645** | **0** | **Both** | **B40:01** | **0.645** |
| 3 | VEEPSSKDVM | CHGA | 121-130 | P10645 | 0 | IFN-α | B40:01/B49:01 | 0.536/1.540 |
| **1** | **GESRSEAL** | **CHGA** | **268-275** | **P10645** | **0** | **IFN-α** | **B40:01** | **0.403** |
| **1** | **GELEQEEERL** | **CHGA** | **323-332** | **P10645** | **0** | **IFN-α** | **B40:01** | **0.174** |
| 2 | REDSLEAGL | CHGA | 399-407 | P10645 | 0 | IFN-α | B40:01 | 0.062 |
| 3 | LEAGLPLQV | CHGA | 403-411 | P10645 | 0 | Both | B49:01/B40:01 | 0.017/0.239 |
| **2** | **AELEKVAHQL** | **CHGA** | **442-451** | **P10645** | **0** | **IFN-α** | **B40:01** | **0.039** |
| 3 | FEGRELLVI | CPE | 83-91 | P16870 | 0 | IFN-α | B49:01/B40:01 | 0.267/0.529 |
| **2** | **KEGGPNNHL** | **CPE** | **203-211** | **P16870** | **0** | **IFN-α** | **B40:01** | **0.091** |
| **4** | **WEDNKNSLI** | **CPE** | **358-366** | **P16870** | **0** | **IFN-α** | **B49:01/B40:01** | **0.262/0.440** |
| **4** | **SESC(+305.07)PVVGM** | **KIF1A** | **923-931** | **Q12756** | **S-glutathionylation** | **IFN-α** | **B40:01/B49:01** | **0.212/0.431** |
| **4** | **GERGFFYTP** | **INS** | **44-52** | **P01308** | **0** | **Both** | **B49:01/B40:01** | **0.412/1.200** |
| **4** | **REAEDLQVGQV** | **INS** | **56-66** | **P01308** | **0** | **Both** | **B49:01/B40:01** | **0.198/0.528** |
| **2** | **AEDLQVGQVEL** | **INS** | **58-68** | **P01308** | **0** | **Both** | **B40:01** | **0.135** |
| **2** | **VELGGGPGAGSL** | **INS** | **66-77** | **P01308** | **0** | **Both** | **B40:01** | **0.327** |
| **2** | **REAEDLQGSL** | **INS-205** | **56-65** | **P01308** | **0** | **IFN-α** | **B40:01/B49:01** | **0.043/0.347** |
| **4** | **AEIPGGPEA** | **PCSK1** | **37-45** | **P29120** | **0** | **IFN-α** | **B49:01/B40:01** | **0.066/0.321** |
| 2 | GEDARGTWTL | PCSK2 | 561-570 | P16519 | 0 | IFN-α | B40:01 | 0.082 |
| **4** | **AERPLNEQI** | **SCG3** | **38-46** | **Q8WXD2** | **0** | **IFN-α** | **B49:01/B40:01** | **0.019/0.134** |
| **4** | **AEDIVHKI** | **SCG3** | **148-155** | **Q8WXD2** | **0** | **IFN-α** | **B49:01/B40:01** | **0.032/0.416** |
| 4 | KEKETLITI | SCG3 | 294-302 | Q8WXD2 | 0 | IFN-α | B49:01/B40:01 | 0.007/0.100 |
| 3 | SEADIQRLL | SCG5 | 36-44 | P05408 | 0 | IFN-α | B40:01/B49:01 | 0.010/0.019 |
| 3 | VEYPAHQAM | SCG5 | 58-66 | P05408 | 0 | IFN-α | B40:01/B49:01 | 0.034/0.076 |
| **3** | **AEFSREFQL** | **SCG5** | **138-146** | **P05408** | **0** | **IFN-α** | **B40:01/B49:01** | **0.016/0.045** |
| **3** | **REFQLHQHL** | **SCG5** | **142-150** | **P05408** | **0** | **IFN-α** | **B40:01** | **0.008** |

**Supplementary Table 4. HLA-B40-restricted candidate epitopes used for screening the reactivity of TCRs and T-cell lines from the islet-infiltrating T cells of HLA-B40$^+$ nPOD T1D organ donors.** TCR reactivity was screened with individual peptides, while T-cell lines were screened using the indicated peptide pools (Fig. 9a-b-c and Supplementary Fig. 9b). Peptides testing positive after deconvolution of individual reactivities (Fig. 9d-e-f) are marked in bold.

| nPOD case / RRID:SAMN# | Negative control peptides |
|---|---|
| 6480 / 15879533 | **Irrelevant allele: HLA-A*02:01**<br>$GAD_{114-123}$: VMNILLQYVV<br>$IA\text{-}2_{797-805}$: MVWESGCTV<br>$IGRP_{265-273}$: VLFGLGFAI<br>$PPI_{15-24}$: ALWGPDPAAA<br>$ZnT8_{186-194}$: VAANIVLTV<br>INS $DRiP_{1-9}$: MLYQHLLPL |
| 6536 / 18242780 | **Irrelevant allele: HLA-B*07:02**<br>$GAD_{3-11}$: SPGSGFWSF<br>$GAD_{100-108}$: ACDGERPTL<br>$GAD_{175-184}$: HPRYFNQLST<br>$GAD_{311-320}$: IPSDLERRIL<br>$GAD_{498-506}$: KPQHTNVCF<br>$GAD_{530-538}$: APVIKARMM<br>$PPI_{8-16}$: LPLLALLAL |
| 6563 / 30386851 | **Irrelevant allele: HLA-B*39:06**<br>$PPI_{5-12}$: MRLLPLLA<br>**Irrelevant HLA-A*02:01-binding peptide**<br>West Nile Virus $PP_{430-438}$: SVGGVFTSV |
| 6566 / 33284286 | **Irrelevant allele: HLA-B*39:06**<br>$PPI_{5-12}$: MRLLPLLA |

**Supplementary Table 5. Negative control peptide pools and individual peptides used for screening the reactivity of T-cell lines from the islet-infiltrating T cells of HLA-B40$^{+}$ nPOD T1D organ donors.** Peptide(s) binding to irrelevant HLA-I alleles (i.e. not expressed by the corresponding donor) or an irrelevant viral peptide binding to the HLA-A*02:01 allele expressed by donor 6563 were used. These experiments are presented in Fig. 9 and Supplementary Fig. 9b.

| Gene | Forward primer | Reverse primer |
|---|---|---|
| *PMSB5* | GCCTTCAAGTTCCGCCAT | TGCCTAGCAGGTATGGGTTG |
| *PMSB6* | CAACCACTGGGTCCTACATCG | GGAAACCGAGCTGGTAGGTG |
| *PMSB7* | AAGTTGCCTTATGTCACCATGG | ATGGCTTCGCTCACCAGAT |
| *PMSB8* | GGCTGTACTATCTGCGAAATGG | AGTCCAGGACCCTTCTTATCCC |
| *PMSB9* | ATGCTGACTCGACAGCCTTT | GAGCAATAGCGTCTGTGGTG |
| *PMSB10* | ACTGCCAAAGAAATGCATCAT | ATCGTTAGTGGCTCGCGTAT |
| *INS* | TTCTACACACCCAAGACCCG | ATTGTTCCACAATGCCACGC |
| *INS* | TGTCCTTCTGCCATGGCCCT | TTCACAAAGGCTGCGGCTGG |
| *CHGA* | CTGAACACAGGCAGCTTTCTA | CAGTCAGGAGTTCTCAGCTTTC |
| *PCKS1* | CATTCTTTGCCTGGTGCACTGTGT | TTGTGGCTGAGAAAGGAGACAGGT |
| *PCSK2* | AACCGTGCCTGAGAGATTCC | TCGGTTGTGAGTGTCAGCAC |
| *SYT4* | CACCAGCCGGGAAGAATTTG | GAAGACCAGGCCAAATGCAC |
| *FTO* | GCATGGCTGCTTATTTCGGG | GGATGCGAGATACCGGAGTG |
| *SOX9* | TTCACCTACATGAACCCCGC | AAGGTCGAGTGAGCTGTGTG |
| *GAPDH* | TCGTGGAAGGACTCATGACC | ATGATGTTCTGGAGAGCCCC |
| *ACTB* | ACTCTTCCAGCCTTCCTTCC | CGTACAGGTCTTTGCGGATG |
| *PPIA* | ATGGCAAATGCTGGACCCAACA | ACATGCTTGCCATCCAACCACT |

**Supplementary Table 6. Primers used to quantify gene expression of constitutive proteasome/immunoproteasome subunits and β-cell identity markers.** These experiments are presented in Supplementary Fig. 3 and Supplementary Fig. 7.

| Antibody [Clone] | Supplier, cat# | RRID | Species, clonality | Staining conditions | 2nd Ab fluorophore | Akoya Biosciences cat# | OPAL conditions |
|---|---|---|---|---|---|---|---|
| HLA-B [HC10] | Origene, AM33035PU-N | AB_2728622 | Mouse, mAb | 1:700 1 h RT | OPAL 520 | FP1487001KT | 1:100 8 min |
| HLA-A/B/C/E [EMR8-5] | Abcam, ab70328 | AB_1269092 | Mouse, mAb | 1:700 1 h RT | OPAL 570 | FP1488001KT | 1:80 10 min |
| HLA-A [ARC0588] | Invitrogen, MA5-35106 | AB_2849011 | Rabbit, mAb | 1:500 1 h RT | OPAL 620 | FP1495001KT | 1:100 12 min |
| INS [ICTABLS] | ThermoFisher, 14-9769-82 | AB_2573014 | Mouse, mAb | 1:1,500 1 h RT | OPAL 690 | FP1497001KT | 1:100 10 min |
| GCG [K79bB10] | Abcam, ab10988 | AB_297642 | Mouse, mAb | 1:800 0.5 h RT | OPAL 780 | FP1501001KT | 1:80 10 min |

**Supplementary Table 7. Abs and conditions used for multiparameter immunofluorescence staining of human pancreas tissues.** These experiments are presented in Fig. 7 and Supplementary Fig. 8. RT, room temperature.
