# Supplementary material for: Interferon-α promotes HLA-B-restricted presentation of conventional and alternative antigens in human pancreatic β-cells
Source: Nat Commun. 2025 Jan 17;16:765. doi: 10.1038/s41467-025-55908-9 (PMC11748642; doi:10.1038/s41467-025-55908-9)
Supplement: Supplementary file 2 — Description of Additional Supplementary Files [file 41467_2025_55908_MOESM2_ESM.pdf]

Description of Additional Supplementary Files

Supplementary Data 1. HLA-I-eluted peptides derived from mRNA splice variants identified in ECN90 β-cells. The first column displays the peptide sequence with, in parenthesis, the mass shift of PTMs found on the aa. PTMs are detailed in the next column and were ultimately defined as likely artifactual. Subsequent columns indicate the condition and the number of replicates in which the peptides were identified; aa length; UniProt accession number of the canonical source protein; gene name and description; whether the source is a granule protein (Y, yes; N, no); predicted HLA-I restriction(s) and corresponding ranking based on NetMHC4.1a (NA, not assigned). The last column indicates whether the peptide sequence was validated by spectral matching (ND, not determined).

Supplementary Data 2. Conventional HLA-I-eluted peptides identified in ECN90 β-cells. The first column displays the peptide sequence. Subsequent columns indicate the condition and the number of replicates in which the peptides were identified; aa length; UniProt accession number; aa position within the source protein (referring to the first source protein listed); gene name and description; whether the source is a granule protein (Y, yes; N, no); predicted HLA-I restriction(s) and corresponding ranking based on NetMHC4.1a (NA, not assigned).

Supplementary Data 3. Post-translationally modified HLA-I-eluted peptides identified in ECN90 β-cells. The first column displays the peptide sequence with, in parenthesis, the mass shift of PTMs found on the aa, with PTMs detailed in the next column. Subsequent columns indicate the condition and the number of replicates in which the peptides were identified; aa length; UniProt accession number; aa position within the source protein (referring to the first source protein listed); gene name, synonyms and description; whether the source is a granule protein; predicted HLA-I restriction(s) and corresponding ranking of unmodified sequences based on NetMHC4.1a; and the final PTM origin assigned as likely artifactual (i.e. found in both the modified and

unmodified synthetic peptide) or likely biological (i.e. found only in the modified synthetic peptide). Y, yes; N, no; NA, not assigned; ND, not determined.

Supplementary Data 4. *Cis*-spliced HLA-I-eluted peptides identified in ECN90 β-cells. The first three columns indicate the source protein (UniProt accession number and gene name) and whether its expression is β-cell-enriched. Subsequent columns indicate predicted HLA-I restriction(s) and corresponding ranking based on NetMHCpan4.1a; the condition in which the peptides were identified; the full peptide sequence; its N-terminal and C-terminal splicing fragments; the forward or reverse splicing direction; and the inter-fragment distance. The last two columns indicate whether the peptide was ultimately validated by spectral matching; and the Ensembl accession number when mRNA splice isoforms were also assigned by the MARS algorithm as an alternative aa sequence match.

Supplementary Data 5. HLA-I-eluted peptides identified in primary human islets. The first column shows the peptide sequence with, in parenthesis, the mass shift of PTMs found on the aa. PTMs are detailed in the next column. Subsequent columns indicate the sample in which the peptides were identified (islet preparation 1 and/or 2); aa length; UniProt accession number of the canonical source protein(s); peptide position in the protein (referring to the first source protein listed); gene name, gene synonyms and description; whether the source protein is a granule protein (Y, yes; N, no); predicted HLA-I restriction(s) and corresponding ranking based on NetMHC4.1a (NA, not assigned). GCG is appended to the list of β-cell-enriched source proteins for comparison, with peptide mapping displayed in Supplementary Figure 10.

Supplementary Data 6. Predicted HLA-A2- and HLA-A3-restricted peptides identified in ECN90 β-cells and primary human islets. The comparison was performed between the conventional peptides eluted from ECN90 β-cells and from the two primary islet samples. For PTMs defined as likely artifactual, the native peptide sequence was retained. From left to right, columns indicated: gene name of the source protein, peptide sequence, the treatment condition of ECN90 β-cells in which the peptide was identified, aa length, peptide position in the source protein (referring to

the first source protein listed); gene synonyms and description; whether the source protein is a granule protein or not; HLA-I restriction(s) and corresponding ranking based on NetMHC4.1a; and the presence in the immunopeptidome of islet sample 1 and/or 2.

Supplementary Data 7. Peptide clusters in ECN90 β-cells exposed to proteasome inhibitors. The identity of the peptides identified in each of the clusters (first column) depicted in Supplementary Fig. 3c is provided, ranked according to cluster number and gene name.

Supplementary Data 8. PTMs identified in the immunopeptidome of ECN90 β-cells. The first two columns indicate the PTM name and mass shift observed in MS. The next three columns list the number of peptides and number of source proteins identified with the indicated PTM, along with their distribution between basal and IFN-α-treated conditions. Specific PTM searches were run for those PTMs meeting our selection criteria (shown in bold), i.e. enrichment in either basal or IFN-α-treated condition (barring tryptophan oxidation to kynurenin) and PTMs unlikely to naturally arise during peptide synthesis or MS acquisition and introducible into synthetic peptides. PTMs detected in very few (<10) peptides were only retained if found in predicted HLA-A2 or HLA-A3 binders. In total, 253 PTM sites on 247 peptides (with 6 peptides carrying two distinct PTMs) were identified. The last column indicates the number of peptides for which the PTM was validated by spectral matching, i.e. a match with the peptide synthesized with the modification, but not in the peptide synthesized in its native form. Given their unlikely artifactual origin, phosphorylated and glutathionylated peptides were not compared to the unmodified sequence. Modified peptides validated as likely biological are highlighted in grey.

Supplementary Data 9. Putative HLA-E*01:01 ligands in the immunopeptidome of ECN90 β-cells. To assign HLA-E*01:01 restriction, a ≥3-fold NetMHC4.1a rank score difference with the second-best allele was used. This is a less stringent criterion than the one used for assigning HLA-E*01:01 restriction in Supplementary Tables 1-4, namely a predicted HLA-E*01:01 restriction without any other HLA-I allele with a ranking score <2. The list includes both conventional peptides and peptides carrying PTMs (listed as native sequences, as all PTMs found on these

peptides were defined as likely artifactual). The first column displays the peptide sequence. Subsequent columns indicate the condition and the number of replicates in which the peptides were identified; aa length; UniProt accession number; aa position within the source protein (referring to the first source protein listed); gene name, synonyms and description; whether the source is a granule protein (Y, yes; N, no); predicted HLA-I restriction(s) and corresponding ranking based on NetMHC4.1a.
