# Supplementary material for: Interferon-α promotes HLA-B-restricted presentation of conventional and alternative antigens in human pancreatic β-cells
Source: Nat Commun. 2025 Jan 17;16:765. doi: 10.1038/s41467-025-55908-9 (PMC11748642; doi:10.1038/s41467-025-55908-9)
Supplement: Supplementary file 12 — Reporting Summary [file 41467_2025_55908_MOESM12_ESM.pdf]

nature portfolio

Corresponding author(s): Roberto MALLONE

Last updated by author(s): Dec 22, 2024

Reporting Summary

Nature Portfolio wishes to improve the reproducibility of the work that we publish. This form provides structure for consistency and transparency in reporting. For further information on Nature Portfolio policies, see our Editorial Policies and the Editorial Policy Checklist.

Statistics

For all statistical analyses, confirm that the following items are present in the figure legend, table legend, main text, or Methods section.

n/a	Confirmed	
☐	☒	The exact sample size (*n*) for each experimental group/condition, given as a discrete number and unit of measurement
☐	☒	A statement on whether measurements were taken from distinct samples or whether the same sample was measured repeatedly
☐	☒	The statistical test(s) used AND whether they are one- or two-sided<br>*Only common tests should be described solely by name; describe more complex techniques in the Methods section.*
☒	☐	A description of all covariates tested
☒	☐	A description of any assumptions or corrections, such as tests of normality and adjustment for multiple comparisons
☐	☒	A full description of the statistical parameters including central tendency (e.g. means) or other basic estimates (e.g. regression coefficient) AND variation (e.g. standard deviation) or associated estimates of uncertainty (e.g. confidence intervals)
☐	☒	For null hypothesis testing, the test statistic (e.g. *F*, *t*, *r*) with confidence intervals, effect sizes, degrees of freedom and *P* value noted<br>*Give P values as exact values whenever suitable.*
☒	☐	For Bayesian analysis, information on the choice of priors and Markov chain Monte Carlo settings
☒	☐	For hierarchical and complex designs, identification of the appropriate level for tests and full reporting of outcomes
☐	☒	Estimates of effect sizes (e.g. Cohen's *d*, Pearson's *r*), indicating how they were calculated

Our web collection on statistics for biologists contains articles on many of the points above.

Software and code

Policy information about availability of computer code

Data collection	Mass spectrometry data was collected with commercial ThermoFisher Fusion Lumos or Bruker timsTOF SCP software.<br>Flow cytometry data was collected with commercial BD FACSDiva v9.0 or Cytek Aurora SpectroFlo v2.2 software.<br>Tissue immunofluorescence images were collected with commercial Vectra Polaris slide scanner software.
Data analysis	Immunopeptidomics data was analyzed using the commercial softwares PEAKS X and PEAKS X Pro (Bioinformatics Solutions). It was subsequently processed with a custom Python code (version 3.7). This code has been deposited in the Zenodo repository under DOI:10.5281/zenodo.14496237 and is available under a GNU GPLv3 open-source license. It also integrates the publicly available software NetMHCpan4.1a. Where specified, Progenesis QI version 2 and the MARS algorithm (Liao et al, Nat Commun 2024) were also used.<br>Gene expression in RNAseq datasets was quantified using Salmon version 0.13.2 and DESeq2 1.24.0 (Patro et al, Nat Methods 2017). The predicted translation products were aligned using the R package Biostrings v2.52.0.<br>Flow cytometry data was analyzed with FlowJo v10.10.<br>Tissue immunofluorescence quantification was performed with Indica HALO image analysis sofware.

For manuscripts utilizing custom algorithms or software that are central to the research but not yet described in published literature, software must be made available to editors and reviewers. We strongly encourage code deposition in a community repository (e.g. GitHub). See the Nature Portfolio guidelines for submitting code & software for further information.

Data

Policy information about availability of data

All manuscripts must include a data availability statement. This statement should provide the following information, where applicable:

- Accession codes, unique identifiers, or web links for publicly available datasets
- A description of any restrictions on data availability
- For clinical datasets or third party data, please ensure that the statement adheres to our policy

The mass spectrometry data generated in this study have been deposited in the ProteomeXchange Consortium via the PRIDE partner repository under PXD045265 (ECN90 β-cells) and PXD045211 (primary human islets). The public RNAseq datasets utilized in this study are available in the GEO repository under GSE148058 (human islets) and GSE201719 (human mTECs). All data are included in the Supplementary Information or available from the authors, as are unique reagents used in this Article. The raw numbers for charts and graphs are available in the Source Data file whenever possible. Raw flow cytometry data and tissue immunofluorescence images can be obtained without restriction from R. Mallone and S.J. Richardson, respectively.

Research involving human participants, their data, or biological material

Policy information about studies with human participants or human data. See also policy information about sex, gender (identity/presentation), and sexual orientation and race, ethnicity and racism.

Reporting on sex and gender	This research involves data and biological material previously collected from human organ donors. As such, it does not qualify as research involving human participants (i.e., no active recruitment for the purpose of this study).<br>Sex was assigned based on self-reporting from available clinical metadata. Details are provided in Supplementary Tables.Sex balance was considered in study design, hence no sex-based analysis was performed. Data disaggregated for sex is reported in the Source Data file.
Reporting on race, ethnicity, or other socially relevant groupings	N/A
Population characteristics	Age, sex, HLA genotype, past diagnosis of type 1 diabetes or not.
Recruitment	N/A
Ethics oversight	All recruited human subjects or next of kin provided written informed consent, without participant compensation. Studies were approved by the relevant Ethics Committees: Ethics Committee of the University of Pisa, Italy and Integrated Islet Distribution Program (IIDP; https://iidp.coh.org/) for human islet donors; West of Scotland Research Ethics Committee, 15/WS/0258 for EADB pancreas tissue sections (https://pancreatlas.org/); University of Florida Health Center Institutional Review Board, #201600029 for nPOD pancreas tissue sections and isolated islets/live pancreas slices to generated T-cell lines (https://npod.org/); Ouest IV/Nantes Ethics Committee, 2021-A01619-32 for peripheral blood mononuclear cells to generate primary human CD8+ T-cell receptor transductants.

Note that full information on the approval of the study protocol must also be provided in the manuscript.

Field-specific reporting

Please select the one below that is the best fit for your research. If you are not sure, read the appropriate sections before making your selection.

[x] Life sciences [ ] Behavioural & social sciences [ ] Ecological, evolutionary & environmental sciences

For a reference copy of the document with all sections, see nature.com/documents/nr-reporting-summary-flat.pdf

Life sciences study design

All studies must disclose on these points even when the disclosure is negative.

Sample size	Sample size was not predetermined but selected based on the observed effect size. Sample size was further increased when data point dispersion was observed to avoid any outlier bias. Sample sizes are deemed sufficient based on the statistical tests detailed in each figure legend.
Data exclusions	Occasional outlier data points were excluded from the analyses and are detailed in the Source Data Excel file.
Replication	All experiments were performed in at least 2 separate occasions, in several instances by different researchers to limit the risk of individual bias/flaw. All attempts at replication were successful.
Randomization	Samples were allocated to experimental groups according to type 1 diabetes status when relevant.

Blinding	Blinding was not possible for most experiments due to the use of individual cell lines.<br>For immunofluorescence staining of pancreas tissues from type 1 diabetic and non-diabetic donors, blinding was not possible due to the distinctive disease histopathology; and not relevant because an automated quantification was performed.

Reporting for specific materials, systems and methods

We require information from authors about some types of materials, experimental systems and methods used in many studies. Here, indicate whether each material, system or method listed is relevant to your study. If you are not sure if a list item applies to your research, read the appropriate section before selecting a response.

Materials & experimental systems

n/a	Involved in the study	
☐	☒	Antibodies
☐	☒	Eukaryotic cell lines
☒	☐	Palaeontology and archaeology
☒	☐	Animals and other organisms
☒	☐	Clinical data
☒	☐	Dual use research of concern
☒	☐	Plants

Methods

n/a	Involved in the study	
☒	☐	ChIP-seq
☐	☒	Flow cytometry
☒	☐	MRI-based neuroimaging

Antibodies

Antibodies used

HLA immunoprecipitation: anti-HLA-A/B/C/E Ab W6/32 (8-16 mg; produced in-house).

Protein synthesis analysis by puromycin incorporation: AF488-coupled anti-puromycin Ab (RRID:AB_2736875; 1:200 for flow cytometry, 1:1,000 for Western blot); APC-coupled anti-HLA-A/B/C/E Ab (RRID:AB_314879; 1:200); anti-proINS Ab (RRID:AB_10949314; 500 ng/mL for immunoprecipitation, 1:1,000 for Western blot); anti-mouse IgG Ab RRID:AB_330924 (1:2,000).

Antibody validation by flow cytometry on HLA-transduced K562 cells: HLA-A clone ARC0588 (RRID: AB_2849011; 1:100) with secondary goat anti-rabbit IgG Ab RRID:AB_2536097 (1:100); HLA-B clone JOAN-1 (RRID:AB_1076708; 1:100) with secondary goat anti-mouse IgG Ab RRID:AB_2536161 (1:100); HLA-C Ab DT-9 (RRID:AB_2739715; 1:50); HLA-A/B/C/E Ab W6/32 (RRID:AB_314873; 1:20).

FLow cytometry on ECN90 beta cells: HLA-A2 clone BB7.2 (RRID:AB_3068052; 1:100) with secondary goat anti-mouse IgG Ab RRID:AB_2832926 (1:200); HLA-B clone JOAN-1 (RRID:AB_1076708; 1:100), HLA-C Ab DT-9 (RRID:AB_2650941; 1:100), HLA-A/B/C/E Ab W6/32 (RRID:AB_314871; 1:100) with secondary goat anti-mouse IgG Ab RRID:AB_2921066 (1:200).

Flow cytometry on primary human islets: BV421-conjugated HLA-A2 clone BB7.2 (RRID:AB_2721522; 1:100); FITC-conjugated HLA-A/B/C/E clone W6/32 (RRID:AB_314872; 1:100); HLA-B clone JOAN-1 (RRID:AB_1076708; 1:100), HLA-C clone DT-9 (RRID:AB_2650941, 1:100), AF488-conjugated goat anti-mouse IgG (RRID:AB_2921066; 1:200); AF647-conjugated anti-insulin (RRID:AB_2739331; 1:100); PE-conjugated anti-glucagon (RRID:AB_2739382; 1:200).

Western blotting on ECN90 beta cells: ARC0588 (HLA-A, RRID:AB_2849011; 1:1,000), horseradish peroxidase-conjugated secondary goat anti-rabbit IgG RRID:AB_2687483 (1:10,000); HC10 (HLA-B, RRID:AB_2728622; 1:1,000), horseradish peroxidase-conjugated secondary horse anti-mouse IgG RRID:AB_330924 (1:10,000); alpha-tubulin Ab RRID:AB_1210457 (1:1,000), horse anti-mouse IgG RRID:AB_330924 (1:10,000).

Tissue immunofluorescence: HLA-B clone HC10 (RRID:AB_2728622; 1:700), OPAL 520 (Akoya Biosciences #FP1487001KT; 1:100); HLA-A/B/C/E clone EMR8-5 (RRID:AB_1269092; 1:700), OPAL 570 (Akoya Biosciences #FP1488001KT; 1:80); HLA-A clone ARC0588 (RRID:AB_2849011; 1:500), OPAL 620 (Akoya Biosciences #FP1495001KT; 1:100); INS clone ICTABLS (RRID:AB_2573014; 1:1,500), OPAL 690 (Akoya Biosciences #FP1497001KT; 1:100); GCG clone K79bB10 (RRID:AB_297642; 1:800), OPAL 780 (Akoya Biosciences #FP1501001KT; 1:80).

Antigen recall on islet-infiltrating CD8+ T cells: for the generation of islet-derived T-cell lines, anti-CD3 (RRID:AB_395736; 2.5 µg/mL); anti-CD28 (RRID:AB_396068; 2.5 µg/mL); anti-Fas (RRID:AB_10596808; 1 µg/mL); anti-PD-1 (RRID:AB_10897007; 1 µg/mL).

All these antibodies are further detailed under the corresponding heading of the Methods section.

Validation

The validation of most antibodies was provided by the manufacturer and by our own data available in published articles: Vecchio et al, Sci Adv 2024; Rachdi et al, eBioMedicine 2023; Quiniou et al, Elife 2023; Mann et al, Front Immunol 2020; Gonzalez-Duque et al, Cell Metab 2018.
For the validation of antibodies against HLA-A, HLA-B, HLA-C and HLA-A/B/C/E, HLA class I-negative K562 cells transduced with different HLA class I alleles were single-stained with these antibodies to confirm specificity. This data is provided in the manuscript.
Further validation of HLA-B specificity for the HC10 antibody (RRID:AB_2728622) is provided in doi:10.4049/jimmunol.171.4.1918.

Eukaryotic cell lines

Policy information about cell lines and Sex and Gender in Research

Cell line source(s)	ECN90 beta-cell line: provided by Human Cell Design; donor sex not available.<br>K562 cell line: provided by ATCC, cat##CCL-243; female donor.<br>5KC murine T-hybridoma cell line: provided by M. Nakayama, Barbara Davis Center, Denver; male mouse donor.
Authentication	The ECN90 beta-cell line was authenticated based on its phenotype (INS+CHGA+) and HLA haplotype.<br>The K562 cell line was authenticated based on the absence of HLA Class I expression.<br>The 5KC cell line was authenticated based on the absence of TCR expression.
Mycoplasma contamination	All cell lines were regularly tested negative for Mycoplasma contamination.
Commonly misidentified lines (See ICLAC register)	No commonly misidentified cell lines were used in the study.

Plants

Seed stocks	N/A
Novel plant genotypes	N/A
Authentication	N/A

Flow Cytometry

Plots

Confirm that:

- [x] The axis labels state the marker and fluorochrome used (e.g. CD4-FITC).
- [x] The axis scales are clearly visible. Include numbers along axes only for bottom left plot of group (a 'group' is an analysis of identical markers).
- [x] All plots are contour plots with outliers or pseudocolor plots.
- [x] A numerical value for number of cells or percentage (with statistics) is provided.

Methodology

Sample preparation	Cell lines were retrieved from culture and washed before staining.<br>Primary human islets were dissociated with TrypLE (ThermoFisher) for 10 min at 37°C prior to staining.
Instrument	BD LSRFortessa flow cytometer. A detailed description of its configuration is provided in Table S6 of doi:10.1016/j.cmet.2018.07.007. A Cytek Aurora flow cytometer was used for some experiments, as detailed in the Methods section.
Software	Data was acquired with BD FACSDiva v9.0 or Cytek Aurora SpectroFlo v2.2 and analyzed with FlowJo 10.10.
Cell population abundance	No sorting was perfomed.
Gating strategy	All experiments gated cells based on physical (FSC/SSC) parameters, doublet exclusion (FSC-H/FCS-A or SSC-W/SSC-H) and viability (Live/DEAD staining).<br>For human islet experiments, beta-cells and alpha-cells were separated based on AF647-INS and PE-GCG staining. This gating strategy can be found in Supplementary Fig. 6c.<br>For cytotoxicity experiments, relevant and irrelevant targets were separated based on prior CFSE vs. CTV staining. T cells were gated based on murine TCR staining. This gating strategy can be found in Supplementary Fig. 9a.<br>Boundaries between positive and negative staining were set based on parallel samples stained with an isotype control antibody or left unstained.

- [x] Tick this box to confirm that a figure exemplifying the gating strategy is provided in the Supplementary Information.
